# Use of Drugs in Clinical Practice and the Associated Cost of Cancer Treatment in Adult Patients with Solid Tumors: A 10-Year Retrospective Cohort Study

**DOI:** 10.3390/curroncol30090580

**Published:** 2023-08-30

**Authors:** Maria-Josep Carreras, Elena Tomás-Guillén, Anna Farriols, Berta Renedo-Miró, Carolina Valdivia, Jana Vidal, Cristina Saura, Joan Carles, Enriqueta Felip, Maria-Queralt Gorgas, Josep Tabernero, Josep Monterde

**Affiliations:** 1 Pharmacy Department, Vall d’Hebron University Hospital, E-08035 Barcelona, Spain; annamaria.farriols@vallhebron.cat (A.F.); berta.renedo@vallhebron.cat (B.R.-M.); carolina.valdivia@vallhebron.cat (C.V.); jana.vidal@vallhebron.cat (J.V.); mariaqueralt.gorgas@vallhebron.cat (M.-Q.G.); 2 Asserta Global Healthcare Solutions, Sant Quirze del Vallés, E-08192 Barcelona, Spain; elena.tomas@asserta.net (E.T.-G.); j.monterde@asserta.net (J.M.); 3 Medical Oncology Department, Vall d’Hebron University Hospital, Vall d’Hebron Institute of Oncology (VHIO), E-08035 Barcelona, Spain; csaura@vhio.net (C.S.); jcarles@vhio.net (J.C.); efelip@vhio.net (E.F.); jtabernero@vhio.net (J.T.)

**Keywords:** cancer, expenditure, antineoplastic agents, solid tumors, supportive care, retrospective cohort study, real word data

## Abstract

Background: Cancer is one of the leading causes of morbidity and mortality in the world. Its growing incidence and prevalence, as well as the advances in diagnostic and treatment tools, motivate an open debate about the economic burden it may place on health systems and have raised concerns about access to this technological innovation. There is a lack of information on the detailed costs of pharmacological treatment of cancer in our health setting. In this context, it is necessary to know the use of drugs in cancer treatment in conditions of real clinical practice. A real-word, evidence-based retrospective cohort study was conducted at Vall d’Hebron University Hospital (VHUH), the largest hospital complex in Catalonia, Spain, in order to determine the use of drugs and the associated cost in real clinical practice for the treatment of solid tumors in adult patients attended at this institution over 10 years (2010–2019). Methods: This was a single-center retrospective cohort study of adult cancer patients attended in clinical practice at the Medical Oncology Department of VHUH between 1 January 2010 and 31 December 2019. Data of prescription, preparation, and cost of antineoplastic treatments were analyzed by pharmacological class (cytotoxic drugs, immunotherapy, targeted therapy, radiopharmaceuticals, and others), by antineoplastic agent, and by type of tumor. The number of patients and the pharmaceutical expenditure corresponding to all these subgroups were recorded. The cost per patient in each tumor location was also calculated. Results: The study population included 13,209 patients with an overall pharmaceutical antineoplastic expenditure of EUR 120,396,097, increasing from 7.67% in relation to the total HUVH pharmaceutical expenditure in 2010 to 12.82% in 2019. By pharmacological class, the specific weight of the cost of targeted therapy is relevant (75.22% of pharmaceutical antineoplastic expenditure, 21.3% of patients) compared to the group of conventional cytotoxics (17.25% of pharmaceutical antineoplastic expenditure, 76.37% of patients), while immunotherapy has represented the largest relative increase, from 5% in 2014 to 12% in 2019. Eight targeted therapy drugs represented 50% of the costs of the targeted therapy drug class (palbociclib, trastuzumab, pertuzumab, bevacizumab, nivolumab, cetuximab, pembrolizumab, and trastuzumab emtansine). Eleven tumor sites accounted for 90% of the expenditure in 71% of all patients. Breast cancer had the highest expenditure during the study period (EUR 34,332,210) and at each individual year. Melanoma showed the highest increase, with 9.7% of total pharmaceutical antineoplastic expenditure in 2019 (2% of patients), representing a paradigm of the rising costs of cancer treatment due to the incorporation of new high-cost therapies. The average annual cost per patient was highly variable depending on the pathology. There was a growing increase in costs per patient in most tumor locations, particularly in patients with melanoma (from EUR 1922 in 2010 to EUR 37,020 in 2019), prostate cancer (from EUR 2992 in 2010 to EUR 14,118 in 2019), and non-small cell lung cancer (from EUR 3545 in 2010 to EUR 8371 in 2019). The relevance of the difference in monthly cost per patient that has been identified for the different intrinsic subtypes in breast cancer patients during 2019 (HER2+ EUR 2661/month, Luminal EUR 881/month, Triple negative EUR 386/month) makes us consider suggesting differentiated reimbursement rates for certain clinical conditions. Finally, support treatment with antiemetic drugs, erythropoietin stimulating agents, granulocyte-colony stimulating factor (G-CSF), and bone resorption inhibitors has involved a cost of EUR 5,751,910, which represents 4.6% of the overall pharmacological cost of cancer treatment. Conclusion: This study provides detailed insights on the oncological pharmaceutical expenditure for the treatment for solid tumors in the VHUH, based on real cost information from our hospital practice and for all antineoplastic therapies and types of solid tumors. This type of information on all the different types of cancer can be useful to better understand the economic burden of the disease and can be decisive for allocating public resources and funds for research, especially in those areas where information is scarce and therefore where further studies are needed. The contribution to knowledge of the cost of oncology therapy is of great value due to its realism and scope.

## 1. Introduction

Cancer is one of the main causes of morbidity and mortality worldwide. In 2020, the Global Cancer Observatory (GLOBOCAN) of the International Agency for Research on Cancer of the World Health Organization reported around 19 million new cancer cases (cumulative incidence: 20.4%) with a mortality of more than 9 million (cumulative incidence: 10.6%) [1]. Also, the number of new cases will increase by 70% in the forthcoming years, and it is projected that there will be approximately 24 million new cancer cases worldwide by 2035 [2]. In Spain, the absolute number of patients diagnosed with cancer has continued to increase for decades, probably in relation to the increase in the general population, aging of the population, exposure to risk factors, and increase of early detection programs in some cancer types [3]. In 2020, a total of 277,394 new cancer cases were reported, with colon and rectum, prostate, breast, and urinary bladder cancers as the most commonly diagnosed tumors [4]. Due to the high prevalence of cancer and based on data of the National Institute of Statistics (INE), tumors still remain as one of the main causes of admission to the hospital [5]. In 2015, malignant tumors were the third cause of hospital admission after cardiovascular and respiratory diseases [6]. Local data referred to Catalonia, an autonomous community in Northeastern Spain with Barcelona as the capital, also showed an increase of 22.5% and 24.5% of cancer cases among men and women, respectively, between 2010 and 2020 [7]. The 5-year relative survival rate for the period 2005–2007 was 54% in men and 62.5% in women [8], which is similar to the median overall survival rates found in the European context [9].

The worldwide cost of antineoplastic therapy and supportive care reached USD 113 billion in 2016, representing an annual increase of 11% compared to the previous year. The United States and the EU5 region (Spain, United Kingdom, Germany, France, and Italy) accounted for 46% and 21% of the global costs, respectively. The annual growth rate of antineoplastic treatment was 8.7% in the years 2011–2016, significantly higher than the 4.9% annual growth recorded for the previous period of 2006–2011. These costs were estimated to reach USD 147 billion in 2021 [10]. A study that estimated the economic burden of cancer in 27 European countries revealed wide differences between countries, with total cancer cost of EUR 126 billion in 2009, and health care accounting for 40% [11]. In Spain, cancer spending accounted for 4% of total healthcare expenditure and 0.86% of the gross domestic product (GDP). Drug spending in the EU as a whole accounted for 27% of the total costs (with variability ranging from 15% to 61%). and in Spain, it was proportionally higher than the average (37%) [11].

Costs of cancer care are major concerns and challenges for any healthcare system [12,13,14,15,16,17,18]. An analysis of health expenditure in the 27 EU member states revealed that higher health expenditures in Western European countries were associated with both increased cancer incidence and decreased cancer mortality as compared with Eastern countries, which was particularly noticeable in breast cancer [19]. A survey of 77 public hospitals conducted by the Spanish Society of Medical Oncology (SEOM) showed an important variability in the different Spanish autonomous regions in the access to 11 new oncology drugs recently approved by the European Medicines Agency (EMA) for the treatment of breast cancer, melanoma, lung cancer, prostate cancer, and supportive care [20]. The inequity was attributed to the existence of a series of binding commissions at different levels, with varying composition and without common criteria, and urged health authorities to work on implementing strategies for the management of oncological diseases that seek to reduce disparities between regions and hospitals [20].

In Catalonia, the Pharmacotherapeutic Harmonization program of the Catalan Healthcare System (CatSalut) guarantees equity in the access to hospital drugs and prescription medicines and improvement in the levels of efficiency, effectiveness, and therapeutic utility based on the principles of rational use, availability, and optimization of resources. In fact, 53% of new therapies evaluated by this program for the period 2010–2017 were oncological agents [21]. On the other hand, one of the objectives of the Catalan Cancer Health Plan 2015–2020 was to perform an annual evaluation of the clinical and variability of use of different oncological medical treatments in the framework of the public healthcare system [22].

There is a lack of information on the detailed costs of the pharmacological treatment of cancer in our health setting. In this context, it is necessary to gather information on the current utilization of drugs in the treatment of cancer in real-world conditions, with detailed data regarding the number and characteristics of treated patients, type of tumors, disease stages, and follow-up, as well as the budgetary impact of treatment-associated expenditure for the system. Real-world evidence (RWE) studies have some strengths and weaknesses but can be useful to describe the treatments in a “clinical practice” population characterized by lower selection bias compared with strict eligibility criteria of randomized controlled clinical trials (RCTs), adding details on the outcome of special patient populations, mainly those underrepresented or excluded from pivotal clinical trials. In addition, RWE could provide data about drug use and cost in different geographical settings and/or economic contexts [23]. The aim of our study was to analyze the use of drugs and the associated costs of cancer treatment in clinical practice in adult patients with solid tumors attended at the Medical Oncology Department of a tertiary care hospital in Barcelona (Spain) over a 10-year period. Complete data of oncological treatments and their corresponding costs in a large population of cancer patients for an extended period of time will provide a close approximation to the actual costs of cancer treatment in the hospital setting in real-world conditions.

## 2. Materials and Methods

### 2.1. Study Design and Participants

This was a single-center retrospective cohort study of adult cancer patients attended in clinical practice at the Medical Oncology Department of Vall d’Hebron University Hospital between 1 January 2010 and 31 December 2019. The primary objective of the study was to assess the pattern of utilization and the costs associated with the use of antineoplastic drugs and supportive therapy in cancer patients treated at the hospital during the study period. The Vall d’Hebron University Hospital is located in Barcelona and is the largest hospital complex in Catalonia (1146 beds, 7814 professionals) and one of the largest in Spain. Its area of influence includes a population of more than 430,000 inhabitants and has an annual budget of EUR 663 million [24]. The Medical Oncology Department integrates the provision of health care and biomedical research activities (preclinical, translational, and clinical) supported by the Vall d’Hebron Institute of Oncology (VHIO). The Pharmacy Department is a cross-sectional service that has three major care units (general area, traumatology and rehabilitation area, and children’s and women’s area) and two satellite pharmacy units: the Hospital Outpatient Prescriptions Unit and the Pharmacy Oncology Unit, which includes various subunits located at the different oncology day hospitals.

For the purpose of the study, data of all consecutive patients aged 18 years or older diagnosed with solid malignant tumors and treated at the Medical Oncology Department between 2010 and 2019, with specific intravenous antineoplastic drugs (including all parenteral intravenous, intramuscular, and subcutaneous routes) or hospital outpatient medicines (specific oral antineoplastic agent and/or supportive therapy), were recorded. Patients included in clinical trials were excluded from the study.

Approval by the Research Ethics Committee was waived due to the healthcare quality of care nature of the study.

### 2.2. Tumor Classification, Active Principles, Therapeutic Groups, and Expenditure

The types of tumors were classified by body location/system according to the classification for adult cancer patients of the National Cancer Institute [25] (Table 1).

For each tumor location, treatments prescribed were grouped by extension of the disease divided into locoregional disease (neoadjuvant and adjuvant treatments) and advanced disease (first line and successive lines for metastatic disease).

The antineoplastic drugs were categorized following the International Common Denomination of Drugs (ICD) or International Non-Proprietary Names (INN) when they existed in marketed specialties according to the database of the Spanish Agency of Medicines and Medical Devices (AEMPS) [26]. Based on the mechanism of action, the following pharmacological classes were established: cytotoxic drugs, immunotherapy, targeted therapy, radiopharmaceuticals, and others.

For each year of the period 2010–2019, all treatments used in the study patients were assessed according to the following therapeutic groups: (a) specific treatments, including intravenous and oral antineoplastic agents; and (b) supportive treatments, including antiemetic drugs, erythropoietin stimulating agents, granulocyte-colony stimulating factor (G-CSF), and bone resorption inhibitors. Data of prescription and preparation of antineoplastic treatments were grouped by pharmacological class, tumor location, active drugs, and extension of the disease. The number of patients and the pharmaceutical expenditure corresponding to all these subgroups were recorded.

### 2.3. Information Sources

The management and treatment of data over 10 years was an extremely complex process. Information sources included different non-integrated software of health data management, such as Sisinf^®^ (Cache for Windows x86-32 2009.1.2 -Build 602- Intersystems), QuimioProcess^®^ (V.1912111511), Silicon^®^ (V.8.5 to 11.2), Lug Traza^®^ (V.2009), and Kiro Oncology^®^ (V.1.1.0.0 to 1.4.3.1.b1-Kiro Soft® V.1.0.1 to 1.1.3), the business intelligence big data of which were then integrated and processed using the Pharmacy Analytics Management (PAM)^®^ (V.2022.1.0) work station (a health analytics tool developed by Asserta Global Healthcare Solutions). PAM^®^ allowed visualization of data in real time and the generation of dynamic reports. A detailed description of the information systems used to retrieve information and management of data is shown in Supplementary S3.

### 2.4. Data Management and Pharmaceutical Expenditure

In order to ensure the quality of the data, cleaning, homogenization/standardization, and enrichment processes were applied. Data enrichment included: (a) assignment of the costs for intravenous antineoplastic treatments based on the prescribed dose and the average annual purchase price of each pharmaceutical specialty and year; (b) assignment of the costs for specific oral antineoplastic treatments based on the prescribed dose; and (c) assignment of the diagnosis of tumor location and type of treatment in patients with specific oral antineoplastic therapy.

Pharmaceutical expenditure was calculated according to the actual price (for each year) of pharmaceutical specialties extracted from the accounting system of the Vall d’Hebron University Hospital, according to the financed price regulated and established by the Ministry of Health in the Interministerial Price Commission for all centers of the Spanish public health system network [27,28], and applying special deductions and payments corresponding to financial agreements established by the Spanish legislation and agreed upon by the Ministry of Health [29,30] and the autonomic health administration of Catalonia [31].

For the cost calculation, only the direct costs of acquiring the pharmaceutical specialties marketed according to the AEMPS (Spanish Agency for Medicines and Health Products) database [26] were taken into account, and indirect non-healthcare costs and intangible costs were not included. On the other hand, the cost of each individualized prescribed dose was considered, including the optimization of vial fractions carried out by the centralized drug preparation unit of the hospital.

The average annual cost per patient of antineoplastic treatments was calculated for each natural year of the study period, analyzing the total expenditure for the annual period and dividing it by the number of patients treated annually. The average cost of the complete treatment per patient throughout their cancer treatment during the study period was also calculated. The following aspects were considered: (1) the number of different patients was calculated by identifying each patient throughout their oncological treatment during the study period, from the beginning to the end; (2) the identification of treatment for each patient was performed using the SAP clinical history number as the identifier for each individual patient; (3) any scheme or sequence of schemes with intravenous or oral cancer drugs used in the same patient, as long as it is the same tumor location, formed part of the same patient’s treatment, but a new tumor location was counted as a different patient; (4) patients who had not completed their treatment by the end of the study and had not received any treatment in the last three months of 2019 were excluded from the analysis; and (5) the total pharmaceutical antineoplastic expenditure for the period 2010–2019 was analyzed and divided by the number of different patients treated in this period. In addition, a special analysis of the costs associated with the treatment of breast cancer and other tumor locations was performed.

### 2.5. Statistical Analysis

Descriptive statistics are presented. In the analysis of the quantitative variables, the sum totals of the values and position measures (central tendency) were used; the quantitative values, calculated as averages, are expressed as the mean and standard deviation (SD) (or median and interquartile range 25th–75th percentile if non-normal distribution of data). For the study of the categorical variables, the absolute number of cases was expressed. For the graphic representation of these variables, relative frequency tables and heat maps were used. Pharmacy Analytics Manager^®^ (V.2022.1.0) was used for analysis.

## 3. Results

### 3.1. Pharmacological Expenditure of Cancer Treatment

The study population included 13,209 patients who received specific antineoplastic treatment for solid tumors during the 10-year study period. The overall pharmaceutical expenditure for antineoplastic drugs was EUR 120,396,097, which accounted for 8.9% of the total pharmaceutical expenditure of the Vall d’Hebron University Hospital and 10.57% of outpatient hospital medicines. As shown in Table 2, the pharmaceutical expenditure for antineoplastic drugs increased over the study period from 7.67% in relation to the total pharmaceutical expenditure in 2010 to 12.82% in 2019.

### 3.2. Expenditure by Pharmacological Classes

In relation to pharmacological class, there has been a substantial increase in the weight of targeted therapy, with 21.3% of patients accounting for 75.22% of total antineoplastic drugs cost as compared with 76.37% of patients treated with conventional cytotoxic agents, which accounted for only 17.25% of the total antineoplastic drugs cost (Table 3). Immunotherapy was the pharmacological class that has experienced a remarkable increase in use, starting in 2012 and growing from 5% of the total antineoplastic drug expenditure in 2014 up to 12% in 2019 (Table 3).

In the group of immunotherapy, the introduction of checkpoint inhibitors began in 2014 with the anti-CTLA-4-antibody ipilimumab followed by anti-PD-1/PD-L1 inhibitors, nivolumab, pembrolizumab, and atezolizumab. The relative contribution of the different drugs to the 2019 antineoplastic drug expenditure was 53% for nivolumab, 30% for pembrolizumab, 11% for atezolizumab, 7% for ipilimumab, and 0% for interferon alpha, cemiplimab, durvalumab, and avelumab.

Targeted therapy has experienced the newest therapeutic incorporations, particularly anti-HER2 and anti-EGFR drugs, antiangiogenic agents, tyrosine kinase inhibitors, mTOR inhibitors, new hormonal agents, and cyclin-dependent kinase inhibitors.

The group of cytotoxic agents showed a proportional decrease in relation to the total antineoplastic drug expenditure. Some cost reductions were related to the end of the exclusivity period granted by the patent and the appearance of generics (e.g., docetaxel in 2011 or capecitabine in 2014). In recent years, some cytotoxic drugs are still representative in the total antineoplastic drug expense, such as pemetrexed (2.74% of total expense in 2019), trabectedin (0.91%), albumin-bound paclitaxel (0.71%), cabazitaxel (0.55%), and eribulin (0.51%).

### 3.3. Expenditure by Antineoplastic Agents

A total of 112 antineoplastic agents were registered, including 46 cytotoxic agents (41.07% of drugs and 17.25% of antineoplastic drug expenditure), 56 targeted therapy drugs (50% drugs and 75.22% of antineoplastic drug expenditure), 8 immunotherapeutic drugs (7.14% of drugs and 6.15% of antineoplastic drug expenditure), and 2 radiopharmaceuticals (1.79% of drugs and 1.38% of antineoplastic drug expenditure). Details of costs by active drugs and year of study are shown in Table S1 of the Supplementary Materials.

In 2019, only eight antineoplastic agents (7.14% of the total) accounted for 50% of the antineoplastic drug expenditure (in decreasing order): palbociclib (9.45%), trastuzumab (8.56%), pertuzumab (7.94%), bevacizumab (7.79%), nivolumab (6.33%), cetuximab (4.85%), pembrolizumab (3.57%), and trastuzumab emtansine (3.21%). On the other hand, of the 34 drugs that accounted for 90% of pharmaceutical antineoplastic expenditure in 2019, 76.5% were targeted therapy agents (72.36% of expenditure). There were only three cytotoxic drugs: pemetrexed (2.74%), trabectedin (0.91%), and albumin-bound paclitaxel (0.71%); almost all immune checkpoint inhibitors, including nivolumab (6.33%), pembrolizumab (3.57%), atezolizumab (1.36%), and ipilimumab; (0.79%), as well as one radiopharmaceutical, ^177^Lu-oxodotreotide (1.32%).

### 3.4. Expenditure by Tumor Location

The pharmaceutical antineoplastic expenditure by tumor location and year are shown in Table S2 of the Supplementary Materials. In 2019, only 11 tumor sites accounted for 90% of the pharmaceutical antineoplastic expenditure in 71% of all patients. These tumor locations, in decreasing order, were as follows: breast, non-small cell lung, melanoma, colon, prostate, kidney, ovary, neuroendocrine, oral and oropharyngeal cavity, soft-tissue sarcoma, and thyroid (Table 4).

**Figure 1:**
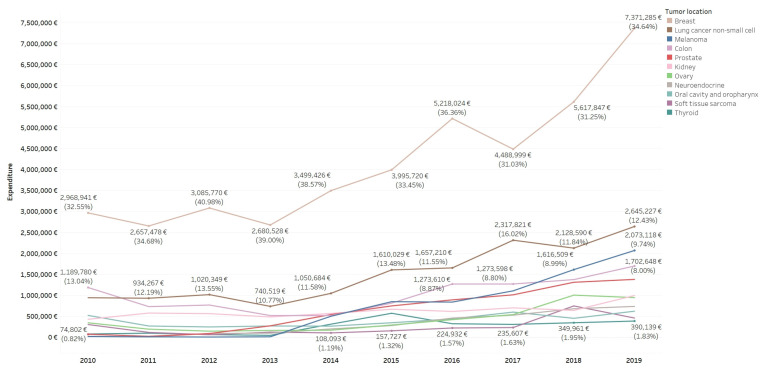
Pharmaceutical antineoplastic expenditure (EUR) (90%) by tumor location and study year.

Changes of pharmaceutical antineoplastic expenditure over the study period by tumor location (Figure 1) showed increases for melanoma (0.23% in 2010 to 9.74% in 2019) and neuroendocrine tumors (0.27% in 2010 to 3.47% in 2019), due to the use of radiopharmaceuticals and targeted therapies, and prostate cancer (0.82% in 2010 to 6.50% in 2019). Other tumors, such as GIST, showed a reduction of expenditure due to the fact that although it incorporated the use of imatinib in 2010 as one of the first targeted therapies, its relative cost has been reducing with the change to generic imatinib and the introduction of other expensive targeted therapies in other pathologies. Malignancies with the highest number of patients did not experience marked changes, except for colon cancer (13.04% in 2010 to 8% in 2019).

Regarding the overall distribution of patients by tumor location, there were 15 tumor sites that accounted 90% of the patients throughout the period 2010–2019, including (in decreasing order): breast cancer (*n* = 3330, 25.20% of patients), non-small cell lung cancer (*n* = 1701, 12.87%), colon cancer (*n* = 1670, 12.64%), and with less weight, oral cavity and oropharynx cancer (5.78%), ovarian cancer (4.81%), rectum (4.60%), exocrine pancreas (4.32%), stomach (3.80%), small-cell lung cancer (3.72%), brain (2.90%), urinary bladder (2.63%), prostate (2.50%), uterine cervix (1.76%), extrahepatic bile duct (1.70%), and soft-tissue sarcoma (1.63%). Table 5 shows the tumor locations accounting for 90% (*) of the pharmaceutical antineoplastic expenditure in the 10-year study period.

**Figure 2:**
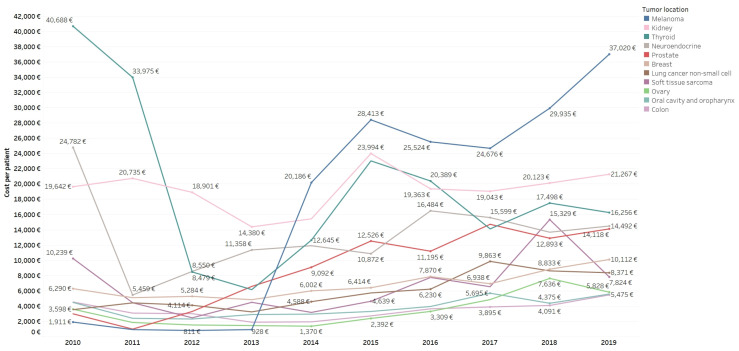
Mean annual cost per patient (EUR) by tumor location (90% of pharmaceutical antineoplastic expenditure) and study year.

There was a growing increase in costs per patient in most tumor locations (Figure 2), particularly in patients with melanoma (variation of 1837% from EUR 1911/patient in 2010 to EUR 37,020/patient in 2019), prostate cancer (variation of 371.86%, from EUR 2992/patient in 2010 to EUR 14,118/patient in 2019), and non-small cell lung cancer (variation of 136.14%, from EUR 3545/patient in 2010 to EUR 8371/patient in 2019). By contrast, GIST (variation of −78.286%, from EUR 20,553/patient in 2010 to EUR 4459/patient in 2019) and brain tumors (variation of −76.75%, from EUR 10,415/patient in 2010 to EUR 2421/patient in 2019) showed decreasing costs per patient.

Heat maps of pharmaceutical antineoplastic expenditure for the complete treatment cost per patient in the period 2010–2019, by tumor location and extension of disease, are shown in Figures S1 and S2 of the Supplementary Materials.

Figure S1. Heat map of expenditure (EUR), cost of complete treatment per patient (EUR ) and number of patients by tumor location for the period 2010–2019.

Figure S2. Heat map of expenditure (EUR), cost of complete treatment per patient (EUR) and number of patients by tumor location and type of treatment for the period 2010–2019.

### 3.5. Breast Cancer

Breast cancer had the highest pharmaceutical antineoplastic expenditure during the 10-year study period and at each individual year. Of a total of 3330 breast cancer patients, identification by molecular subtype (luminal [RH+ HER2], human epidermal growth factor receptor 2 HER2+ [RH+/− HER2+], triple negative [RH− HER2−], according to modified classification of the European Society of Medical Oncology [ESMO] [32]) was available in 2308 patients (69.3%). For this population, the total pharmaceutical expenditure was EUR 34,332,210. The distribution by molecular intrinsic subtypes is shown in Table 6.

In 2019, in the HER2+ population, 90% of the expense was related to the use of three anti-HER2 drugs, trastuzumab (41%), pertuzumab (40%), and trastuzumab emtansine (16%), followed by palbociclib, docetaxel, and lapatinib, each with 1% of the expense. In 2010, there were only two anti-HER2 drugs, with a marked difference in expense in favor of trastuzumab (86.86%) followed by a remarkable decrease of lapatinib (8.92%).

In patients with the luminal subtype in 2019, the predominant drugs were cyclin inhibitors palbociclib (67.44%) and ribociclib (4.90%), as well as bevacizumab (15.11%), followed by everolimus (3.10%). In 2010, however, cytotoxic drugs such as docetaxel (64.08%), liposomal doxorubicin (18.49%), and vinorelbine (5.17%) were the predominant antineoplastic agents.

In the subgroup of the triple-negative subtype, in 2019, pharmaceutical antineoplastic expenditure was mostly associated with the use of the antiangiogenic drug bevacizumab (43.93%) and the chemotherapeutic drugs liposomal doxorubicin, eribulin, albumin-bound paclitaxel, docetaxel, vinorelbine, which together accounted for 48.9% of antineoplastic expenditure. In 2010, there was a reduced use of bevacizumab (4.95%), and the expenditure of docetaxel (59.3%) was greatly marked by its high price, followed by liposomal doxorubicin (13.16%) and vinorelbine (12.72%).

The monthly costs per patient for the different years and according to the three molecular subtypes are shown in Table 7. There were marked differences for each subtype, with particularly noticeable increases in the expenditure for the HER2+ and luminal subtypes between 2010 and 2019.

For the year 2019, the overall cost of breast cancer treatment was EUR 4,259,045 in patients with HER2+ cancer, EUR 2,005,265 in those with luminal cancer, and EUR 143,413 in those with triple-negative tumors. The corresponding monthly costs per patient were EUR 2661, EUR 881, and EUR 386, respectively.

### 3.6. Other Tumors

Six other malignancies, including non-small cell lung cancer, melanoma, colon cancer, prostate, kidney cancer, and epithelial ovarian carcinoma, together with breast cancer, accounted for 80% of the pharmacological antineoplastic expenditure during the study period. The mean (SD) cost per patient was EUR 8848 (EUR 24,372) for non-small cell lung cancer, EUR 38,218 (EUR 53,175) for melanoma, EUR 6193 (EUR 13,500) for colon cancer, EUR 19,326 (EUR 27,771) for prostate, EUR 44,221 (EUR 53,656) for kidney cancer, and EUR 6703 (EUR 19,285) for ovarian cancer.

In patients with non-small cell lung cancer, 95.84% of the pharmaceutical antineoplastic expenditure was explained by four therapeutic groups: immunotherapy (33.74%), drugs targeting EGFR-activating mutations (28.36%), pemetrexed (22.16%), and ALK tyrosine kinase inhibitors (11.58%). Treatment of melanoma in 2019 was represented by two therapeutic groups (99.86% of expenditure), including the combination of BRAF serine-threonine kinase inhibitors and MEK kinases (53.17%) and immune checkpoint inhibitors (46.69%). In colon cancer patients, pharmaceutical antineoplastic expenditure in 2019 included monoclonal anti-EGFR antibodies (53.78%), anti-angiogenic agents (31.92%), BRAF-MEK inhibitors (7.35%), the multi-kinase inhibitor regorafenib (1.19%), and cytotoxic agents (4.57%). In patients with prostate cancer, 99.9% of expenditure in 2019 was associated with the new anti-androgens enzalutamide (44.7%) and abiraterone (35.42%), the radiopharmaceutical radium-223 dichloride (8.62%), and the cytotoxic taxanes cabazitaxel (8.55%) and docetaxel (2.69%). In patients with kidney cancer, the three types of drugs that accounted for 100% of the expenditure included tyrosine kinase inhibitors (65.65%), immunotherapy (26.24%), and the mTOR inhibitor temsirolimus (8.11%). Finally, in women with epithelial ovarian tumors, 99.77% of the expenditure was related with the use of poly (ADP-ribose) polymerase (PARP) inhibitors (53.49%), the anti-angiogenic agent bevacizumab (41.39%), and cytotoxic agents (4.89%).

### 3.7. Supportive Therapy

In relation to antiemetics, the total expenditure during 2010–2019 was EUR 1,071,755, with annual variations ranging from −45.78% to 26.15%, and a decrease in the expenditure from 2010 to 2019 of −76.6%. These changes have been strongly influenced by the introduction of generic versions of anti-5HT-3 drugs, the incorporation of anti-NK1 inhibitors, and the most recent introduction of a generic version of aprepitant.

Erythropoietin-stimulating agents accounted for a total expenditure of EUR 1,461,773, with reductions throughout the study period in the number of patients treated (−10.87% in 2019 compared to 2010), associated total costs (−66.57%) and average cost per patient (−62.50%).

The pharmaceutical expenditure for the use of G-CSF was EUR 2,081,554 (36.19% of all supportive treatment expenditure). Filgastrim was the agent used in 81.66% of patients (30.91% of the expenditure), and pegfilgastim was used in 18.34% of patients (69.09% of the expenditure). The average cost per treated patient was EUR 105 in 2019, with important differences between filgastim and pegfilgastim (EUR 94 vs. EUR 1129 per patient).

The bone resorption inhibitors used were zoledronic acid and denosumab. The expenditure of zoledronic acid for the period 2011−2019 was EUR 640,306, with an average cost per patient ranging from EUR 1480 in 2011 to EUR 51 in 2019. The use of denosumab started in 2013, accounting for EUR 496,521 in the period 2013–2019. The mean cost per patient decreased from EUR 2600 in 2013 to EUR 1500 in 2019.

A summary of the total pharmaceutical expense of antineoplastic agents and supportive therapy over the 10-year study period is shown in Table 8.

## 4. Discussion

The present retrospective cohort study provides extensive information of detailed costs of cancer treatment in a population of adult patients with solid tumors attended in a reference Medical Oncology Department of one of the largest hospitals in Spain in the framework of a public healthcare system. Moreover, the use of cancer treatment and associated costs in clinical practice conditions have been evaluated by tumor location, by antineoplastic agent, and by the mean annual and the complete treatment costs per patient, but more remarkably, data have been consecutively recorded over a 10-year period. To our knowledge, no study with these characteristics has been carried out previously.

Between 2010 and 2019, the total pharmaceutical expenditure of oncological treatment in 13,209 different adult patients with solid tumors was EUR 126,148,006, 95.4% of which corresponded to the costs of antineoplastic drugs for a total of EUR 120,396,096. We found very variable annual expenditure from −15.40% to 32.01%, and this is a relevant finding of the study. Two studies on the economic burden of drug cancer costs in Spain in 2009 and 2015, respectively, showed annual variations around 1% [11,33], which is lower than the annual rate of 6.12% found in our study for the 2010–2015 period. These differences may be explained by methodological characteristics of these studies (drug costs based on hospital and community pharmacy sales, inclusion of hormone therapy, use of 2015 cost data based on estimations from 2017 adjusted by annual inflation) and the use different expenditure information sources rather than the homogeneity of data sources in the present study. The total expenditure of antineoplastic drugs is a significant proportion of the total pharmaceutical expenditure of the VHUH (8.9% of global pharmaceutical expenditure and 10.57% of outpatient hospital drugs). A strategy for reducing costs is price negotiation, and it is important to know the cost of therapies in clinical practice in order to prioritize those therapeutic strategies identified with low or uncertain clinical benefit in this negotiation, since some studies have not identified an association between the costs of treatment for solid tumors and the clinical benefit measured by the ASCO-Value Framework (ASCO-VF) and the ESMO-Magnitude of Clinical Benefit Scale (ESMO-MCBS) scales, both in the USA and in European countries [34].

Costs of treatment included 75.22% for targeted therapies (21.3% of patients), 17.25% for cytotoxic drugs (76.37% of patients), 6.15% for immunotherapy (1.91% of patients), and 1.38% for radiopharmaceuticals (0.38% of patients). In fact, from 2010 to 2019, there was an increase of 132.7% in the annual expenditure, with a substantial increase of immunotherapy (from 5% to 12%) and a decrease of cytotoxic agents (from 31% to 9%). In a study carried out in Lebanon of the costs of oncology drugs between 2014 and 2016, the expenditure increased by 27% after the introduction of immunotherapy in 2015 [35]. In a study of the strategies to control rising spending on cancer drugs, Bach [36] analyzed how the monthly costs of antineoplastic drugs at the time of their commercialization have multiplied since before the introduction of targeted therapies. The decrease in expenditure of cytotoxic drugs is mostly related to their decrease in price rather than a reduction in their clinical use. In a study of drugs approved by the FDA for solid tumor treatment between 2000 and 2015 of four therapeutic groups (agents targeting oncogenes, anti-angiogenics, immunotherapy, and chemotherapy) included in 74 studies, there were minimal differences in median monthly costs among the therapeutic groups, except for chemotherapy with a significantly lower cost [37]. This is consistent with the fact that cytotoxic drugs in our study contributed very little to spending (9% in 2019) due to their low price compared to innovative therapies, except for three cytotoxic drugs that are among the 34 drugs that generate 90% of the spending: pemetrexed, trabectedin, and albumin-bound paclitaxel, all of which are high-priced because they still have exclusivity due to an unfinished patent period.

On the other hand, the introduction of generic drugs has been widely adopted in cytotoxic drugs, with remarkable decreases in the prices of pharmaceutical specialties. This has led to a clear decremental variation in drug expenditure on topotecan (−98.40%), epirubicin (−95.79%), docetaxel (−85.22%), bleomycin (−73.84%), irinotecan (−77.97%), oxaliplatin (−77.67%), temozolomide (−71.98%), and doxorubicin (−71.62%), without substantially changing the prescription pattern and number of treated patients.

Regarding the 34 antineoplastic agents that accounted for 90% of the drug expenditure in 2019, 76.5% were targeted therapy drugs (72.69% of expenditure). There is a notable incremental variation in the spending generated by emerging drugs in recent years, such as cyclin inhibitors (palbociclib, ribociclib), new anti-HER2 agents (pertuzumab, trastuzumab emtansine), and new immune checkpoint inhibitors (nivolumab, pembrolizumab). In a Lebanese study [35], trastuzumab was the costliest drug in the 2014–2016 period, being replaced in the ranking by pembrolizumab, which, together with nivolumab in third place, accounted for 19% of the annual budget for only 3% of patients. Data of our study for 2019 show the role of palbociclib (9.49% of expenditure); anti-HER2 drugs with 19.8% (trastuzumab 8.60%, pertuzumab 7.97%, trastuzumab emtansine 3.23%); bevacizumab with 7.83%; PD-1/PD-L1 inhibitors 11.32% (nivolumab 6.36%, pembrolizumab 3.59%, atezolizumab 1.37%); and anti-EGFR with 7.27% (cetuximab 4.88%, panitumumab 2.39%). The only biological antineoplastic that had a biosimilar in our country during the period of study, trastuzumab, was introduced to our center in 2019. In this year, 188 breast cancer patients were treated with trastuzumab, with the original 79% of them causing 96.1% of spending on trastuzumab, and 21% with the biosimilar causing only 3.9% of trastuzumab spending. A greater reduction in costs is expected due to the expansion of its use and the incorporation of the biosimilar bevacizumab, marketed in Spain since July 2020.

A relevant contribution of the study is the analysis of pharmaceutical expenditure by 42 tumor locations. A total of 11 tumor locations accounted for 90% of the expenditure in 71% of the patients. In decreasing order, these included breast, non-small cell lung, melanoma, colon, prostate, kidney, ovary, neuroendocrine, oral and oropharyngeal cavity, softtissue sarcoma, and thyroid.

Breast cancer stands out every year as the cancer tumor that involves the highest expenditure affecting the largest number of patients. It should be noted that the prevalence of the HER2+ subgroup under treatment (33%) is probably overestimated, compared to the 25.2% reported in other series [38], due to the identification of anti-HER2-treated patients from the pool of patients without intrinsic molecular subtyping at the time of data integration and the concentration of long-term survivors with continuous anti-HER therapy in the longitudinal analysis. The relevance of the difference of monthly costs according to intrinsic molecular subtypes (HER2+ EUR 2661, luminal EUR 881, and triple negative EUR 386) indicates that billing rates should be adapted to subtypes of breast cancer patients.

The present results of breast cancer costs agreed with the study by Bermejo de las Heras et al. [39] of an incidence-based cost-of-illness model in a cohort of metastatic breast cancer patients followed over 5 years. The economic burden differed by HER2 and HR status, with HER2+/HR +patients having the highest per patient costs. The costs for active treatment and follow-up for 5 years in this study (between EUR 20,366 and EUR 150,131 depending on the subtype, with an average of EUR 58,664) were higher than those obtained in our study for the complete 10-year follow-up cohort (HER2+ EUR 36,671, luminal EUR 4312, triple negative EUR 3035), which can be explained by the different methods used to estimate sample characteristics and treatments applied (a theoretical incidence model and a survey of experts) and their costs (based on catalog prices, which are much higher than the actual prices at our institution). Other studies carried out in Spain have reported similar data [40,41]. Capri et al. [42] conducted a study on breast cancer in a population sample of 12,580 patients between 2007 and 2011, based on real clinical practice data from the cancer registry of the Agency for Health Protection of the Province of Milan, showing a mean treatment cost per patient of EUR 8780, with a large variability and dispersion of results among the 71 centers included in the registry.

Melanoma was the third tumor type (in 2019) in terms of its contribution to the total expenditure (9.74%), while it ranks 14th in the number of treated patients (2.02%). The mean cost per patient in 2019 was EUR 37,020. Melanoma is a paradigmatic example of the rising cost of cancer treatment due to the incorporation of new high-cost therapies, as shown in a systematic review of nine studies on the cost-effectiveness of treatments for advanced melanoma with the introduction of therapeutic innovations with BRAF and MEK inhibitors and immunotherapy [43]. This review reported significant differences in the average costs per patient of treatments with cytotoxic agents temozolomide (EUR 6902 in 1999), dacarbazine (EUR 3697 in 1999), or with new therapies vemurafenib (EUR 49,938 in 2013), dabrafenib-trametinib (EUR 194,876 in 2015), and nivolumab-ipilimumab (EUR 259,293 in 2015). In a study of the cost of illness of melanoma in Europe, new treatment options, in particular, costly drugs, are expected to raise expenditure. Although costs per patient can be considerable and vary markedly depending on the healthcare system, prevention and early detection strategies are crucial to reduce its global burden [44].

Non-small cell lung cancer accounted for 12.43% of expenditure in 11.41% of patients in 2019. Also, the mean annual cost per patient in 2019 was EUR 8371, with a mean cost of complete treatment per patient of EUR 10,004 for the advanced disease. These costs are lower than those obtained by González García et al. [45] based on a cost-efficacy mathematical model of first-line treatment of advanced disease in the pre-immunotherapy era, with EUR 15,594, EUR 19,942, and EUR 36,095 for chemotherapy with bevacizumab-cisplatin-gemcitabine, cisplatin-pemetrexed, and bevacizumab-carboplatin-paclitaxel, respectively. Also, a 30% reduction in acquisition costs was estimated for pemetrexed due to the launch of generic medications [45].

Treatment of colorectal cancer was associated with 9.44% of expenditure for 15.45% of patients during 2019, with mean annual costs for colon and rectal cancer treatment per patient of EUR 5475 and EUR 2626, respectively. Our data are consistent with a study based on clinical practice and real costs of hospital care for a historical cohort of 699 patients with colorectal cancer attended at an acute-care teaching hospital in Barcelona between 2000 and 2006 [46]. The cost of drugs (including day hospital costs, antineoplastics, and antiemetics) was identified as one of the main components of the total cost, with more weight in the advanced stages. During the study period, only the cytotoxic drugs oxaliplatin, irinotecan, fluorouracil, and capecitabine, and a as directed therapy, cetuximab, were used, and the average cost of treatment was EUR 5027, also with great data dispersion. This cost was estimated for the first 5 years from diagnosis and did not include longer-term follow-up [46]. Another retrospective observational study based on clinical practice in a public hospital in the Basque Country [47] described, in a sample of 529 patients with colorectal cancer registered between 2010 and 2013, an average cost of chemotherapy treatment per patient very similar to ours, in localized disease of EUR 1033.2 (EUR 1165 for localized colon and EUR 567 for localized rectum in our study throughout the 10-year period), and in the metastatic disease of EUR 12,789 (EUR 8413 for advanced colon and EUR 7041 for advanced rectum in our study). Other studies in our environment showed higher mean costs when calculated according to laboratory sale prices [48].

The supportive therapy accounted for a total cost of EUR 5,751,910 throughout the study period (4.6% of the total pharmaceutical expenditure for the treatment of solid tumors). The percentage of costs related to supportive therapy showed a marked decrease over the years from 10% in 2010 to 1.4% in 2019. The supportive treatment analyzed has been mainly that of drugs directly related to the management of the adverse effects of antineoplastic treatment or complications of the oncological disease. Chronic multimorbidity in cancer patients represents a high burden of long-term pharmacological treatments (e.g., antihypertensives, platelet aggregation inhibitors, anticoagulants, statins, oral antidiabetics, etc.), and the duration of these treatments has been reported very frequently until the end of life [49]. Also, strategies for the deprescription of potentially inappropriate drugs at the end of life have been described [50]. This aspect has not been analyzed in our study. In the same way, the variation in the use of supportive therapy throughout the patient’s life course has not been analyzed.

The objective of the study was to evaluate the pharmaceutical cost represented by oncological drugs for the treatment of solid tumors used in day hospital, outpatient, and in-patient settings. We analyzed what part this pharmaceutical cost represents with respect to the total pharmacological costs of the center to determine the economic relevance of this therapeutic area (oncology) with respect to the total cost of all therapeutic areas [51]. Due to the size of the study, it involved the integration and analysis of a hug volume of data over 10 years and retrospectively, which makes it difficult to have data on other aspects of costs in a large and complex center such as the Vall d’Hebron University Hospital; this may be considered a limitation of the scope of our analysis.

## 5. Conclusions

During the study period of 2010–2019, 13,209 different adult patients with solid tumors received specific antineoplastic treatment, which amounted to a pharmaceutical expenditure of EUR 120,396,096. Targeted therapy accounted for 75% of the drug expenditure, and eight antineoplastic agents (palbociclib, trastuzumab, pertuzumab, bevacizumab, nivolumab, cetuximab, pembrolizumab, and trastuzumab emtansine) were associated with 50% of the expenditure. Breast cancer was the malignancy with the highest drug costs (34.6% in 2019), and melanoma showed the highest increase, with 9.7% of total pharmaceutical antineoplastic expenditure in 2019 for only 2% of patients, representing a paradigm of the rising costs of cancer treatment due to the incorporation of new high-cost therapies. Robust data on pharmaceutical expenditure for treatment of different types of neoplasms, based on real clinical practice, are essential for a better understanding of the economic burden of cancer; to rationalize the allocation of public resources and funds for research, especially in those areas where information is scarce; and to serve as a basis for the comparison with other studies.

## Figures and Tables

**Table 1:** Cancer types by body location.

Tumor Location	
Adrenal	Neuroendocrine
Anal	Oral cavity and oropharynx
Basal cell skin cancer	Osteosarcoma
Bile duct	Other cutaneous tumors
Brain	Ovarian
Breast	Pancreas endocrine
Colon	Pancreas exocrine
Endometrium	Penis
Esophagus	Primary unknown
Ewing sarcoma	Prostate
Extrahepatic bile duct	Rectum
Gastrointestinal stromal tumors (GIST)	Rhinopharynx/cavum
Hepatic carcinoma	Soft-tissue sarcoma
Kaposi sarcoma	Squamous cell skin cancer
Kidney	Stomach
Larynx/hypopharynx	Testicular germinal
Lung non-small cell	Thymoma
Lung small cell	Thyroid
Melanoma	Urinary bladder
Merkel carcinoma	Uterine cervix
Mesothelioma	

**Table 2:** Pharmaceutical expenditure of the Vall d’Hebron University Hospital for 2010–2019.

Year	In-Patient Medicines	Outpatient Hospital Medicines	Total Pharmaceutical Expenditure *	Antineoplastic Drugs Oncology Department #	% of the Total Pharmaceutical Expenditure	% of the Outpatient Hospital Medicines
2010	€21,763,858	€97,474,270	€119,238,129	€9,144,426	7.67	9.38
2011	€18,147,037	€95.550,281	€113,697,318	€7.736,424	6.80	8.10
2012	€15,847,622	€95,933873	€111,781,495	€7,548,927	6.75	7.87
2013	€15,602,975	€96,196,464	€111,799,439	€6,873,817	6.15	7.15
2014	€16,918,317	€101,346,166	€118,264,483	€9,073,832	7.67	8.95
2015	€20,280,225	€132,033,927	€152,314,152	€11,943,892	7.84	9.05
2016	€21,781,823	€131,303,912	€153,085,735	€14,351,319	9.37	10.93
2017	€22,263,436	€125,493,008	€147,756,444	€14,465,977	9.79	11.53
2018	€27,886,002	€128,115,605	€156,001,607	€17,976,077	11.52	14.03
2019	€30,346,269	€135,619,362	€165,965,631	€21,281,403	12.82	15.69
**Total**	**€210,837,564**	**€1,138,066,868**	**€1,349,904,433**	**€120,396,097**	**8.92**	**10.57**

* Total pharmaceutical expenditure: in-patient medicines + outpatient hospital medicines; # This expenditure includes both in-patient and outpatient hospital antineoplastic medicines.

**Table 3:** Expenditure and number of patients by pharmacological classes.

Year	Expenditure No. Patients	Cytotoxic Drugs	Immunotherapy	Targeted Therapy	Radiopharmaceuticals	Other	Total Antineoplastic Drugs	% Change Previous Year
2010	Expenditure	€2,849,013		€6,295,413			€9,144,426	
	Patients	1720	1	487			1941	
2011	Expenditure	€1,948,767		€5,787,658			€7,736,424	−15.40
	Patients	1647	5	377			1837	
2012	Expenditure	€1,856,463	€4423	€5,688,042			€7,548,927	−2.44
	Patients	1771	5	386			1964	
2013	Expenditure	€1,768,568	€10,155	€5,095,094			€6,873,817	−8.95
	Patients	1811	5	385			2008	
2014	Expenditure	€1,867,964	€450,580	6,755,288			€9,073,832	32.01
	Patients	1804	13	477			2067	
2015	Expenditure	€2,144,778	€685,798	€9,113,317			€11,943,892	31.63
	Patients	1911	47	594			2244	
2016	Expenditure	€2,246,494	€593.732	€11,146,186	€394,908		€14,351,319	20.17
	Patients	2020	49	695	20		2391	
2017	Expenditure	€1,988,765	€1,311003	€10,766,721	€399,352	€136	€14,465,977	0.80
	Patients	1969	65	718	17	3	2376	
2018	Expenditure	€2,116,242	€1,798,999	€13,598,269	€462,037	€530	€17,976,077	24.26
	Patients	2045	96	889	17	5	2543	
2019	Expenditure	€1,985083	€2,577,195	€16,317,770	€400,730	€626	€21,281,403	18.40
	Patients	2141	124	1038	19	4	2772	

**Table 4:** Tumor locations accounting for 90% (*) of pharmaceutical antineoplastic expenditure in 2019.

Tumor Location	% Total Expenditure	% Total Patients	Accumulated Expenditure (Pareto)
Breast *	34.64	26.32	34.64
Lung cancer non-small cell *	12.43	11.41	47.07
Melanoma *	9.74	2.02	56.81
Colon *	8.00	11.23	64.81
Prostate *	6.50	3.54	71.31
Kidney *	4.70	1.70	76.01
Ovary *	4.46	5.88	80.47
Neuroendocrine *	3.47	1.84	83.94
Oral cavity and oropharynx *	2.94	4.04	86.88
Soft-tissue sarcoma *	2.17	2.13	89.05
Thyroid *	1.83	0.87	90.88
Rectum	1.44	4.22	92.33
Pancreas exocrine	1.35	4.19	93.67
Stomach	1.22	3.00	94.90
Brain	1.22	3.86	96.11
Uterine cervix	0.80	1.55	96.91
Gastrointestinal stromal tumors (GIST)	0.80	1.37	97.71
Larynx/hypopharynx	0.39	0.43	98.10
Other	0.35	0.00	98.44
Urinary bladder	0.32	1.88	98.77
Basal cell skin cancer	0.24	0.07	99.01
Other cutaneous tumors	0.16	0.18	99.17
Unknown primary tumor	0.15	0.14	99.33
Hepatic carcinoma	0.14	0.14	99.47
Extrahepatic bile duct	0.12	2.24	99.59
Anal	0.11	0.32	99.70
Endometrium	0.10	1.12	99.80
Lung cancer small cell	0.06	3.00	99.86
Ewing sarcoma	0.04	0.07	99.90
Testicular germinal	0.03	0.25	99.93
Esophagus	0.02	1.16	99.95
Rhinopharynx/cavum	0.02	0.40	99.97
Kaposi sarcoma	0.01	0.04	99.98
Thymoma	0.01	0.11	99.99
Osteosarcoma	0.00	0.18	99.99
Mesothelioma	0.00	0.14	99.99
Pancreas endocrine	0.00	0.07	99.99
Gallbladder	0.00	0.11	100.00
Penis	0.00	0.07	100.00
Adrenal	0.00	0.04	100.00
Squamous cell skin cancer	0.00	0.04	100.00

**Table 5:** Tumor locations accounting for 90% (*) of patients (2010–2019).

Tumor Location	Patients	% Total Patients	Accumulated (Pareto)
Breast *	3330	25.20	25.20
Lung cancer non-small cell *	1701	12.87	38.07
Colon *	1670	12.64	50.71
Oral cavity and oropharynx *	764	5.78	56.49
Ovary *	635	4.81	61.30
Rectum *	608	4.60	65.90
Pancreas exocrine *	571	4.32	70.22
Stomach *	502	3.80	74.02
Lung cancer small cell *	491	3.72	77.74
Brain *	383	2.90	80.63
Urinary bladder *	347	2.63	83.26
Prostate *	330	2.50	85.76
Uterine cervix *	232	1.76	87.51
Extrahepatic bile duct *	225	1.70	89.22
Soft-tissue sarcoma *	216	1.63	90.85
Endometrium	206	1.56	92.41
Esophagus	195	1.48	93.89
Melanoma	188	1.42	95.31
Other	163	1.23	96.54
Kidney	142	1.07	97.62
Neuroendocrine	129	0.98	98.59
Larynx/hypopharynx	88	0.67	99.26
Gastrointestinal stromal tumors (GIST)	81	0.61	99.87
Testicular germinal	76	0.58	100.45
Thyroid	74	0.56	101.01
Mesothelioma	74	0.56	101.57
Unknown primary tumor	51	0.39	101.95
Anal	46	0.35	102.30
Pancreas endocrine	43	0.33	102.63
Osteosarcoma	29	0.22	102.85
Rhinopharynx/cavum	29	0.22	103.07
Ewing sarcoma	25	0.19	103.26
Kaposi sarcoma	18	0.14	103.40
Gallbladder	14	0.11	103.51
Hepatic carcinoma	13	0.10	103.61
Thymoma	12	0.09	103.70
Penis	10	0.08	103.78
Other cutaneous tumors	5	0.04	103.82
Basal cell skin cancer	4	0.03	103.85
Adrenal	4	0.03	103.88
Total	13,214		

There are patients with more than one tumor site.

**Table 6:** Pharmaceutical antineoplastic expenditure in the population of breast cancer patients grouped by molecular intrinsic subtypes during the study period 2010–2019.

Molecular Intrinsic Subtypes	Pharmaceutical Expenditure	% Expenditure	Patients	% Patients	Median Cost per Patient (25th–75th Percentile)
HER2+	€28,126,333	82	767	33	€25,105 (€18,115 to €34.058)
Luminal	€5,161,780	15	1197	52	€198 (€135 to 3348)
Triple negative	€1,044,097	3	344	15	€314 (€154 to 3051)

**Table 7:** Average monthly pharmaceutical antineoplastic expenditure per breast cancer patient (EUR) by intrinsic molecular subtypes during the 2010–2019 study period.

Intrinsic Molecular Subtype	Year	Pharmaceutical Expenditure	Patients	Cost per Patient per Month
HER2+	2010	€1,604,633	118	€2042
	2011	€1,830,649	129	€2094
	2012	€2,161,834	149	€2108
	2013	€1,945,750	148	€1901
	2014	€2,420,214	159	€2306
	2015	€2,981,295	190	€2120
	2016	€4,059,094	198	€2500
	2017	€3,302,505	194	€2402
	2018	€3,561,315	191	€2620
	2019	€4,259,045	216	€2661
Luminal	2010	€122,325	51	€557
	2011	€56,774	66	€210
	2012	€46,715	48	€257
	2013	€155,623	139	€262
	2014	€328,188	216	€273
	2015	€271,211	214	€304
	2016	€428,177	255	€317
	2017	€518,371	275	€332
	2018	€1,229,130	266	€784
	2019	€2,005,265	317	€881
Triple negative	2010	€36,943	17	€421
	2011	€12,391	20	€168
	2012	€41,988	11	€757
	2013	€64,363	52	€255
	2014	€121,186	70	€317
	2015	€160,960	83	€328
	2016	€154,562	70	€443
	2017	€163,439	69	€328
	2018	€144,850	72	€489
	2019	€143,413	78	€386
Total	2010–2019	€34,332,210	2308	€1222

**Table 8:** Summary of the total pharmaceutical expense of antineoplastic drugs and supportive therapy for each study year.

Treatment	Study Year										
	2010	2011	2012	2013	2014	2015	2016	2017	2018	2019	Total
Antineoplastic agents											
Intravenous	€6,565,263	€4,464,740	€4,695,722	€4,436,132	€5,770,959	€7,596,964	€9,769,375	€9,475,022	€11,252,776	€12,666.317	€76,693,270
Oral	€2,579,163	€3,271,684	€2,853,205	€2,437,685	€3,302,873	€4,346,928	€4,581,945	€4,990,955	€6,723,301	€8,615,087	€43,702,826
Total antineoplastic agents	€9,144,426	€7,736,424	€7,548,927	€6,873,817	€9,073,832	€11,943,892	€14,351,320	€14,465,977	€17,976,077	€21,281,404	€120,396,096
Supportive therapy											
Antiemetics	€252,598	€149,146	€80,865	€102,012	€97,625	€86,005	€110,673	€73,862	€59,853	€59,115	€1,071,755
Erythropoietins	€182,782	€261,803	€220,567	€132,728	€105,447	€140,141	€126,266	€142,798	€88,147	€61,093	€1,461,773
G-CSF factors	€582,602	€336,822	€377,331	€92,995	€139,564	€199,158	€207,005	€69,604	€32,554	€43,919	€2,081,554
Bone resorption inhibitors	-	€195,329	€224,430	€120,942	€36,513	€61,983	€121,613	€116,241	€116,079	€143,697	€1,136,827
Total Supportive therapy	€1,017,982	€943,100	€903,193	€448,677	€379,149	€487,287	€565,557	€402,505	€296,634	€307,825	€5,751,910
TOTAL	€10,162,408	€8,679,524	€8,452,120	€7,322,494	€9,452,981	€12,431,179	€14,916,877	€14,868,482	€18,272,711	€21,589,229	€126,148,006

## Data Availability

The study data are available from the corresponding author upon request.

## Supplementary Materials

The following supporting information can be downloaded at: https://www.mdpi.com/article/10.3390/curroncol30090580/s1, Table S1: Pharmaceutical expenditure (€) by antineoplastic agents and study year (2020–2019). Table S2: Pharmaceutical expenditure (€) by tumor location and study year (2020–2019). Supplementary S3 includes the description of the sources of information and details of the management of data. Figure S1. Heat map of expenditure (€), cost of complete treatment per patient (€) and number of patients by tumor location for the period 2010–2019. Figure S2. Heat map of expenditure (€), cost of complete treatment per patient (€) and number of patients by tumor location and type of treatment for the period 2010–2019.
