# Supplementary material for: Use of Drugs in Clinical Practice and the Associated Cost of Cancer Treatment in Adult Patients with Solid Tumors: A 10-Year Retrospective Cohort Study
Source: Curr Oncol. 2023 Aug 30;30(9):7984–8004. doi: 10.3390/curroncol30090580 (PMC10528466; doi:10.3390/curroncol30090580)
Supplement: Supplementary file 1 [file curroncol-30-00580-s001.zip › Supplementary S3 revised.pdf]

**Supplementary S3: information systems used to obtain and manage data.**

During the 10-year period of the study at the Vall d'Hebron University Hospital, different information systems have been used to manage clinical processes and the economic management of drugs in the treatment of oncological tumors. Information sources included different non-integrated softwares of health data management, such as Sisinf® (Cache for Windows x86-32 2009.1.2 -Build 602- Intersystems), QuimioProcess® (V.1912111511), Silicon® (V.8.5 to 11.2), Lug Traza® (V.2009), and Kiro Oncology® (V.1.1.0.0 to 1.4.3.1.b1-Kiro Soft® V.1.0.1 to 1.1.3), the business intelligence big data of which were then integrated and processed using Pharmacy Analytics Management (PAM)® (V.2022.1.0) work station (a health analytics tool developed by Asserta Global Healthcare Solutions). PAM® allowed visualization of data in real time and generation of dynamic reports.

Briefly Sisinf® and QuimioProcess® are designed for the integral management of different phases of the process of use of antineoplastic drugs, including prescription, scheduling and management of oncology day hospitals agendas, validation, preparation and administration of antineoplastic drugs, and quality and quantity control and traceability of all steps. Lug Traza® only for preparation phase. These programs are interfaced with various information systems such as the patient admission registry of SAP® and the analytical data of the patient (SAP®: Systems Applications and Products in Data Processing). Silicon® integrated in the information systems platform (ARGOS project) and SAP Ecofin economic-financial system of the Catalan Health Institute (ICS) was used for electronic prescription and electronic recording of in-patients dispensing and drug administration and outpatient hospital medicines, as well as for recording the consumption and generation of invoices for all antineoplastic treatment (intravenous or oral) and supportive care treatment, used for the reimbursement of outpatient hospital medicines by the CatSalut (Catalan Health System). Lug Traza® (Lug Health Technology) until May 2017, and since then QuimioProcess®, offers qualitative and quantitative control and traceability of preparation of intravenous antineoplastic treatment. Finally, Kiro Oncology® is a robotic system for the preparation of intravenous antineoplastic treatments with automated qualitative, quantitative and traceability control of the processes, and interfaced with Sisinf® and QuimioProcess® programs.
