# Supplementary material for: Use of Drugs in Clinical Practice and the Associated Cost of Cancer Treatment in Adult Patients with Solid Tumors: A 10-Year Retrospective Cohort Study
Source: Curr Oncol. 2023 Aug 30;30(9):7984–8004. doi: 10.3390/curroncol30090580 (PMC10528466; doi:10.3390/curroncol30090580)
Supplement: Supplementary file 1 [file curroncol-30-00580-s001.zip › Supplementary Table S1 revised.pdf]

**Supplementary Table S1.** Pharmaceutical expenditure (EUR) by antineoplastic agent and study year (2020-2019)

| Antineoplastic agent | 2010 | 2011 | 2012 | 2013 | 2014 | 2015 | 2016 | 2017 | 2018 | 2019 |
|---|---|---|---|---|---|---|---|---|---|---|
| Palbociclib | | | | | | | - | - | €919,166 | €2,020,361 |
| Trastuzumab | €1,434,704 | €1,601,506 | €2,044,395 | €1,874,171 | €1,917,997 | €2,271,900 | €2,436,444 | €2,127,679 | €1,972,386 | €1,830,909 |
| Pertuzumab | | | | €32,925 € | €273,281 € | €323,231 € | €832,319 € | €707,468 € | €1,211,167 € | €1,696,546 € |
| Bevacizumab | €1,690,995 | €584,211 | €491,192 | €490,827 | €651,137 | €825,308 | €1,042,487 | €962,766 | €1,398,494 | €1,665,917 |
| Nivolumab | | | | | | - | €225,556 | €749,262 | €984,779 | €1,353,132 |
| Cetuximab | €862,905 | €668,708 | €671,912 | €543,353 | €521,992 | €716,767 | €764,049 | €1,058,344 | €1,076,506 | €1,037,699 |
| Pembrolizumab | | | | | | - | €175,503 | €386,566 | €438,448 | €764,055 |
| Trastuzumab-DM1 | | | | - | €49,408 | €305,346 | €713,887 | €432,871 | €393,072 | €686,316 |
| Dabrafenib | | | | | €13,997 | €10,407 | €185,597 | €220,879 | €500,892 | €685,548 |
| Olaparib | | | | | | - | €137,769 | €439,172 | €609,353 | €627,487 |
| Enzalutamide | | | - | - | | €34,095 | €253,810 | €423,741 | €476,438 | €618,405 |
| Pemetrexed | €389,913 | €364,703 | €426,757 | €387,717 | €420,029 | €654,729 | €631,572 | €595,601 | €497,812 | €586,054 |
| Sunitinib | €424,228 | €730,087 | €618,961 | €386,242 | €422,574 | €556,183 | €315,306 | €347,448 | €391,653 | €544,455 |
| Abiraterone | | | €91,527 | €254,905 | €461,164 | €569,748 | €384,394 | €318,928 | €606,733 | €524,219 |
| Panitumumab | €77,041 | €52,932 | €52,821 | €14,389 | €117,471 | €307,632 | €720,710 | €363,880 | €253,536 | €507,592 |
| Everolimus | - | €41,853 | €19,773 | €59,158 | €139,421 | €172,442 | €273,211 | €434,339 | €618,939 | €420,570 |
| Osimertinib | | | | | | | €28,704 | €525,611 | €390,014 | €362,430 |
| Trametinib | | | | | - | - | €35,733 | €111,963 | €237,069 | €329,618 |
| Carbozantinib | | | | | | €53,002 | €22,996 | €23,460 | €123,553 | €329,615 |
| Atezolizumab | | | | | | | | | €23,119 | €291,302 |
| $^{177}$Lu oxodotreotide | | | | | | | €189,280 | €218,400 | €305,760 | €281,466 |
| Alectinib | | | | | | | | - | €67,258 | €200,249 |
| Trabectedine | €249,722 | €74,799 | €110,759 | €131,924 | €68,565 | €115,062 | €154,425 | €126,593 | €238,343 | €195,448 |
| Lenvatinib | | | | | | €6708 | | €62,416 | €87,367 | €190,330 |
| Vemurafenib | | | | | €59,232 | €105,946 | €223,059 | €123,616 | €151,302 | €174,392 |
| Ribociclib | | | | | | | | | €56,106 | €172,439 |
| Ipilimumab | - | - | | | €441,313 | €682,403 | €162,673 | €175,175 | €345,633 | €168,706 |
| Pazopanib | | €5500 | €128,428 | €163,126 | €284,491 | €257,548 | €284,120 | €233,085 | €123,858 | €168,325 |
| Erlotinib | €600,833 | €608,698 | €516,585 | €296,391 | €387,043 | €462,514 | €322,127 | €186,142 | €153,086 | €158,744 |
| Paclitaxel albumin | €43,323 | | €71,346 | €88,978 | €151,965 | €136,562 | €125,958 | €131,935 | €201,365 | €151,803 |
| Ramucirumab | | | | | | - | - | €88,309 | €68,949 | €137,743 |
| Olaratumab | | | | | | | | | €419,196 | €127,797 |

| Antineoplastic agent | 2010 | 2011 | 2012 | 2013 | 2014 | 2015 | 2016 | 2017 | 2018 | 2019 |
|---|---|---|---|---|---|---|---|---|---|---|
| Afatinib | | | | | €1322 | €123,162 | €148,118 | €104,504 | €79,252 | €126,787 |
| Sorafenib | €261,410 | €207,774 | €50,936 | €51207 | €108,218 | €217,118 | €168,693 | €187,718 | €293,551 | €123,706 |
| $^{223}$Ra dichloride | | | | | | | €205,628 | €180,952 | €156,277 | €119,264 |
| Cabazitaxel | | | - | €23,310 | €78,296 | €142,266 | €53,965 | €88,736 | €100,549 | €118,336 |
| Docetaxel | €771,197 | €153,469 | €49,814 | €19,047 | €16,003 | €13,437 | €12,275 | €6011 | €5974 | €113,965 |
| Aflibercept | | | | | €26,995 | €80,568 | €113,694 | €202,616 | €237,591 | €113,447 |
| Gefitinib | | | €70,110 | €23,612 | €79,112 | €102,318 | €125,804 | €132,793 | €102,151 | €109,689 |
| Eribulin | | €8459 | | €16,400 | €211,598 | €191,592 | €333,879 | €258,519 | €158,398 | €108,200 |
| Temsirolimus | €133,652 | €119,692 | €104,209 | €120,985 | €120,601 | €81,752 | €105,540 | €76,851 | €42,719 | €106,961 |
| Crizotinib | | | | €34,840 | €175,024 | €238,296 | €309,504 | €201,767 | €165,150 | €101,120 |
| Vinorelbine | €167,774 | €175,501 | €88,647 | €73,218 | €80,078 | €73,397 | €81,518 | €100,678 | €104,023 | €100,869 |
| Vismodegib | | | | | | €153,920 | €106,322 | €98,811 | €30,073 | €98,809 |
| Vandetanib | | | | €19,219 | €198,977 | €238,096 | €143,290 | €85,281 | €41,299 | €€96,537 |
| Doxorubicin liposomal | €197,867 | €227,348 | €257,322 | €163,971 | €229,461 | €248,644 | €209,064 | €138,957 | €186,664 | €96,117 |
| Temozolomide | €277,075 | €292,043 | €141,252 | €78,368 | €55,410 | €37,612 | €49,642 | €37,115 | €52,427 | €77,635 |
| Encorafenib | | | | | | | | | | €66,561 |
| Irinotecal peg lip* | | | | | | | - | - | - | €57,646 |
| Gemcitabine | €131,674 | €81,839 | €65,187 | €85,445 | €28,162 | €35,163 | €29,715 | €25,525 | €37,209 | €51,467 |
| Doxorubicin peg lip* | €115,276 | €99,505 | €21,998 | €176,238 | €144,981 | €194,925 | €290,588 | €153,496 | €210,038 | €51,065 |
| Binimetinib | | | | | | | | | | €42,048 |
| Regorafenib | | | - | - | - | €23,475 | €29,761 | €28,523 | €52,509 | €41,489 |
| Paclitaxel | €54,303 | €63,658 | €69,636 | €44,144 | €50,950 | €36,287 | €39,188 | €34,176 | €38,031 | €38,663 |
| Imatinib | €623,656 | €822,882 | €643,884 | €574,734 | €545,662 | €584,900 | €595,213 | €178,921 | €154,566 | €35,017 |
| Carboplatin | €21,639 | €23,125 | €20,186 | €19,243 | €19,077 | €32,240 | €27,959 | €24,694 | €30,711 | €33,867 |
| Cobimetinib | | | | | | - | - | €12,323 | €31,055 | €32,534 |
| Lapatinib | €151,388 | €269,233 | €164,176 | €153,724 | €185,879 | €127,759 | €90,799 | €76,471 | €14,361 | €32,299 |
| Trifluridine/tipiracil | | | | | | - | - | €68,410 | €59,001 | €30,155 |
| Capecitabine | €2394 | €2974 | €306,712 | €271,062 | €92,823 | €31,355 | €29,762 | €95,766 | €29,065 | €28,276 |
| Streptozocin | €8948 | €8377 | €19,467 | €36,110 | €91,627 | €7587 | €2929 | | €29,076 | €26,372 |
| Raltitrexed | €40,988 | €43,950 | €26,833 | €19,937 | €25,091 | €22,709 | €24,483 | €22,249 | €29,266 | €23,752 |
| Fluorouracil | €18,361 | €14,384 | €10,181 | €11,045 | €10,635 | €20,858 | €23,535 | €26,306 | €24,901 | €23,435 |
| Ifosfamide | €4202 | €3084 | €8850 | €8958 | €12,340 | €25,459 | €5553 | €20,083 | €21,579 | €15,368 |
| Irinotecan | €67,783 | €61,063 | €30,206 | €15,951 | €13,542 | €17,611 | €15,363 | €16,530 | €18,240 | €14,932 |
| Axitinib | | | | - | €12,588 | €161,376 | €195,675 | €107,726 | €8224 | €13,413 |

| Antineoplastic agent | 2010 | 2011 | 2012 | 2013 | 2014 | 2015 | 2016 | 2017 | 2018 | 2019 |
|---|---|---|---|---|---|---|---|---|---|---|
| Calcium folinate | €36,996 | €29,217 | €20,731 | €26,621 | €13,383 | €15,196 | €18,717 | €14,461 | €12,908 | €12,673 |
| Oxaliplatin | €53,326 | €35,877 | €28,083 | €19,585 | €10,347 | €11,461 | €12,877 | €13,054 | €11,202 | €11,910 |
| Niraparib | | | | | | | | - | - | €9491 |
| Actinomycin D | €1976 | | €916 | €2068 | | €1641 | €3528 | €2030 | €8072 | €8702 |
| Cisplatin | €9887 | €12,986 | €9559 | €7860 | €7810 | €10,094 | €8899 | €7048 | €8065 | €8133 |
| Abemaciclib | | | | | | | | | - | €7891 |
| Cyclophosphamide | €2971 | €3467 | €4033 | €3220 | €3400 | €3498 | €3516 | €3051 | €2700 | €7617 |
| Doxorubicin | €22,279 | €21,034 | €16,076 | €7002 | €7643 | €2072 | €2571 | €7133 | €6582 | €6322 |
| Ceritinib | | | | | | | - | - | | €5051 |
| Etoposide | €5245 | €4849 | €5018 | €4861 | €6010 | €6382 | €5800 | €3547 | €3976 | €3905 |
| Nintedanib | | | | | | | €27,252 | €79,613 | €38,414 | €3777 |
| Lomustine | | | €42 | | €1869 | €5826 | €6176 | €10,731 | €10,658 | €2641 |
| Fotemustine | €10,992 | €13,841 | €3990 | €4047 | €4209 | €8220 | €2689 | €3037 | €1509 | €2470 |
| Topotecan | €103,962 | €83,182 | €16,234 | €1322 | €1513 | €480 | €373 | €11,817 | €28,014 | €1660 |
| Dacarbazine | €503 | €1499 | €782 | €1128 | €758 | €284 | €187 | €231 | €1009 | €1280 |
| Mitomycin | €239 | €574 | €768 | €532 | €665 | €641 | €624 | €415 | €864 | €928 |
| Procarbazine | | | - | | - | €1089 | €1820 | €3606 | €3244 | €853 |
| Epirubicin | €15,102 | €24,346 | €18,633 | €13,517 | €1743 | €2479 | €1664 | €1561 | €893 | €635 |
| Bleomycin | €1995 | €1356 | €1458 | €816 | €1177 | €764 | €2291 | €592 | €832 | €522 |
| Vincristine | €379 | €57 | €173 | €272 | €170 | €229 | €341 | €638 | €511 | €419 |
| Mitoxantrone | €235 | | | | | | | | €187 | €201 |
| Methotrexate | €185 | €1340 | €209 | €562 | €2379 | €947 | €22 | €30 | €1093 | €101 |
| Vinblastine | | | | €56 | €8 | | | | | €52 |
| Vinflunine | €20,223 | €15,088 | | | | | | | | |
| Thalidomide | €1346 | | | | | | | | - | |
| Nilotinib | €10,384 | | | | | | | | | |
| Mercaptopurine | | | | | €118 | €692 | | | | |
| Interferon-alpha2b | | - | €4423 | €10,155 | €9267 | €3394 | | | | |
| Dexrazoxane | | | | | | | | | €251 | |
| Dasatinib | | | - | - | - | - | €8788 | | | |
| Carmustine | | €1704 | €4602 | €4032 | €4129 | €35,284 | €33,026 | €2813 | | |
| Avelumab | | | | | | | | | €7020 | |

*peg lip: pegylated liposomal.
