# Supplementary material for: Use of Drugs in Clinical Practice and the Associated Cost of Cancer Treatment in Adult Patients with Solid Tumors: A 10-Year Retrospective Cohort Study
Source: Curr Oncol. 2023 Aug 30;30(9):7984–8004. doi: 10.3390/curroncol30090580 (PMC10528466; doi:10.3390/curroncol30090580)
Supplement: Supplementary file 1 [file curroncol-30-00580-s001.zip › Supplementary Table S2 revised.pdf]

Supplementary Table S2. Pharmaceutical expenditure (EUR) by tumor location and study year (2010-2019)

Tumor location	2010	2011	2012	2013	2014	2015	2016	2017	2018	2019
Breast	€2,968,941	€2,657,478	€3,085,770	€2,680,528	€3,499,426	€3,995,720	5,218,024	€4,488,999	€5,617,847	€7,371,285
Lung cancer non-small	€946,485	€934,267	€1,020,349	€740,519	€1,050,684	€1,610,029	€1,657,210	€2,317,821	€2,128,590	€2,645,227
Melanoma	€21,016	€14,853	€8916	€14,848	€504,657	€852,375	€842,296	€1,110,430	€1,616,509	€2,073,118
Colon	€1,189,780	€734,506	€772,037	€521,907	€522,712	€817,529	€1,273,610	€1,273,598	€1,378,816	€1,702,648
Prostate	€74,802	€26,780	€92,474	€276,563	€545,533	€751,587	€895,605	€1,015,597	€1,315,128	€1,383,520
Kidney	€432,113	€580,586	€567,035	€488,923	€571,175	€671,818	€619,628	€704,596	€643,921	€999,534
Ovary	€349,051	€197,145	€148,987	€166,134	€172,678	€296,656	€423,570	€544,245	€1,008,017	€950,039
Neuroendocrine	€24,782	€10,917	€94,047	€102,219	€202,538	€282,669	€461,547	€530,352	€684,125	€739,101
Oral/oropharynx	€524,853	€272,032	€249,891	€272,748	€268,850	€350,021	€442,835	€603,648	€455,043	€625,116
Soft tissue sarcoma	€307,176	€120,013	€64,280	€126,003	€108,093	€157,727	€224,932	€235,607	€751,144	€461,601
Thyroid	€81,376	€101,924	€67,832	€49,311	€316,128	€575,684	€326,216	€310,590	€349,961	€390,139
Rectum	€75,870	€43,373	€119,647	€159,145	€103,611	€151,033	€323,753	€207,215	€277,552	€307,256
Pancreas exocrine	€73,812	€45,140	€43,502	€66,156	€88,923	€118,136	€80,205	€111,138	€153,189	€286,688
Stomach	€52,469	€69,711	€81,988	€129,278	€74,082	€118,647	€112,551	€174,977	€180,686	€259,801
Brain	€354,109	€235,264	€160,539	€190,298	€125,840	€203,873	€309,088	€157,580	€176,514	€259,095
Uterine cervix	€33,920	€18,704	€2129	€1742	€42,912	€28,338	€35,534	€45,002	€293,987	€170,633
GIST	€513,333	€806,305	€693,251	€617,521	€584,736	€626,675	€672,509	€298,806	€308,016	€169,451
Larynx/hypopharynx	€9397	€5146	€69	€28,937	€66,941	€59,614	€37,709	€70,511	€142,335	€82,046
Urinary bladder	€55,682	€36,183	€16,229	€13,372	€4810	€7,862	€10,168	€1,155	€12,538	€68,569
Basal cell skin cancer						€69,264	€56,561	€12,888	€25,777	€51,553
Other skin tumors										€34,368
Primary unknown	€689	€1596	€6643	€2313	€1542	€14,922	€1005	€1519	€11,157	€32,913
Hepatic carcinoma	€12,221	€40,195	€3404	€17,930	€6082		€56	€10,446	€33,209	€30,690
Extrahepatic bile duct	€6454	€8184	€9700	€8604	€3776	€5595	€16,178	€20,214	€14,757	€26,294
Anal	€553	€475	€325	€693	€300	€334	€345	€5042	€27,942	€20,268
Endometrium	€22,251	€5807	€2917	€5061	€3311	€4371	€3330	€5403	€21,148	€18,243
Lung small cell	€47,939	€58,233	€22,059	€9047	€6418	€8504	€14,159	€15,100	€27,579	€9579
Ewing sarcoma	€3820	€548	€6197	€1801	€5540	€7446	€13,511	€5524	€8765	€6415
Testicular germinal	€3540	€2793	€2939	€1231	€3871	€7934	€7469	€799	€1707	€3586
Esophagus	€10,388	€5053	€3734	€1897	€2706	€3159	€1683	€3578	€3371	€2553
Rhinopharynx/cavum	€2				€132	€217	€1031	€759	€3183	€1270
Kaposi sarcoma	€21,229	€13,157	€5101	€15,907	€1227	€8328	€11,199	€18,761	€634	€877

Tumor location	2010	2011	2012	2013	2014	2015	2016	2017	2018	2019
Thymoma		€219	€489	€480	€103	€58	€172		€89	€656
Osteosarcoma	€3137	€2621	€371	€1878	€3439	€4161	€2674	€2946	€4506	€489
Mesothelioma	€109,975	€102,611	€65,634	€65,949	€35,053	€41,968	€78,716	€62,452	€70,466	€339
Pancreas endocrine	€20,803	€51,262	€96,739	€75,198	€139,639	€86,006	€71,242	€8950	€18,645	€268
Gallbladder	€6094			€1,177	€370	€97			€100	€234
Penis	€23,837	€146		€14	€343	€97	€162	€76	€140	€191
Adrenal							€345	€974	€9495	€55
Squamous cell skin										€48
Merkel carcinoma									€7020	
Not available	€738,292	€429,979	€4657	€7773	€5648	€2608	€69,209	€72,076	€144,595	€74,066

GIST: gastrointestinal stromal tumors; data without information for tumor location are shown as “not available”, and represent real pharmaceutical expenses accounting only 1.29% of total expenses for the overall 2010-2019 period and only 0.003% for the year 2019.
