# Supplementary figures and images for: Use of Drugs in Clinical Practice and the Associated Cost of Cancer Treatment in Adult Patients with Solid Tumors: A 10-Year Retrospective Cohort Study
Source: Curr Oncol. 2023 Aug 30;30(9):7984–8004. doi: 10.3390/curroncol30090580 (PMC10528466; doi:10.3390/curroncol30090580)

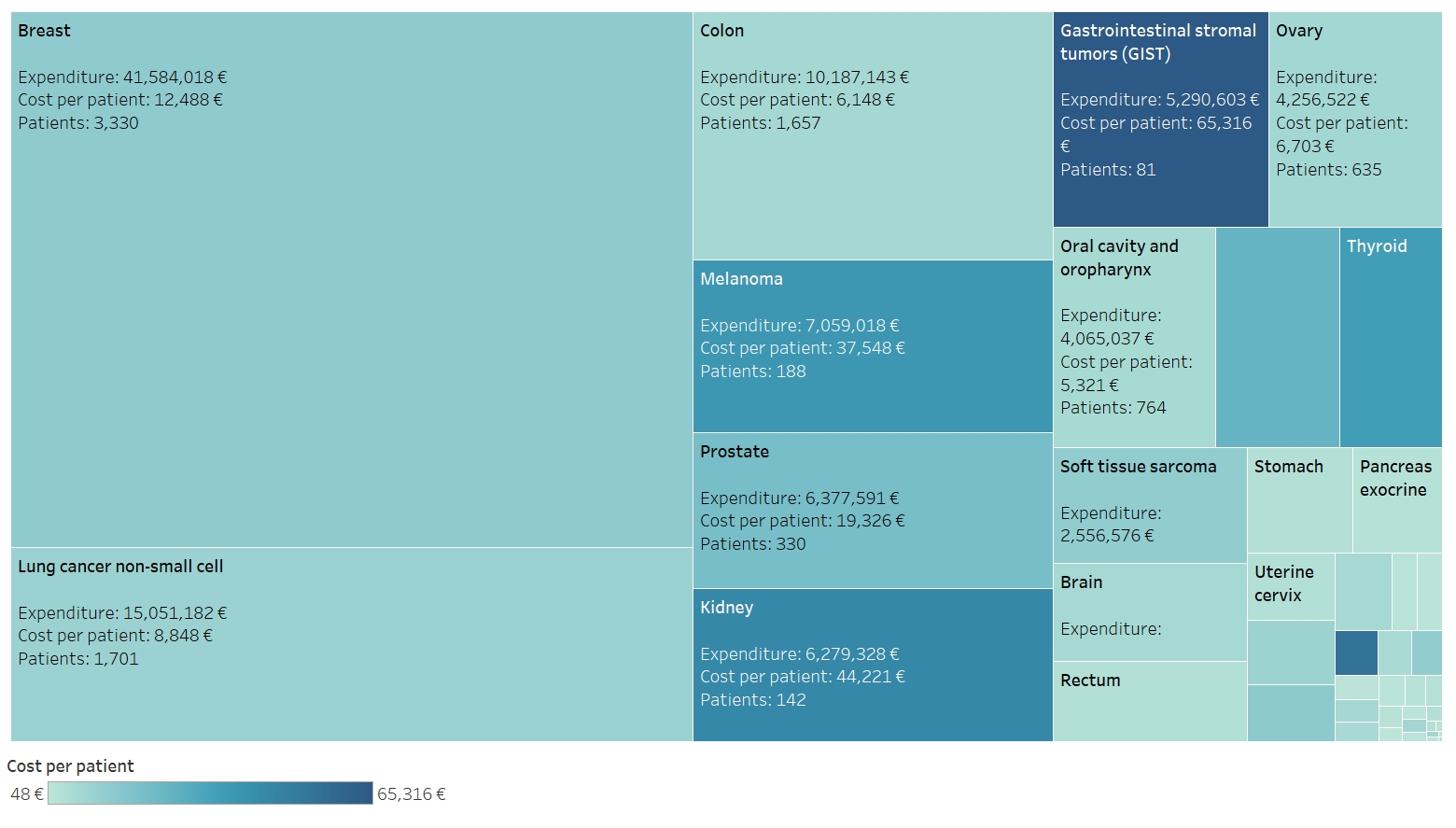

Supplement: Supplementary file 1 [file curroncol-30-00580-s001.zip › Supplementary Figure S1 revised.jpg]

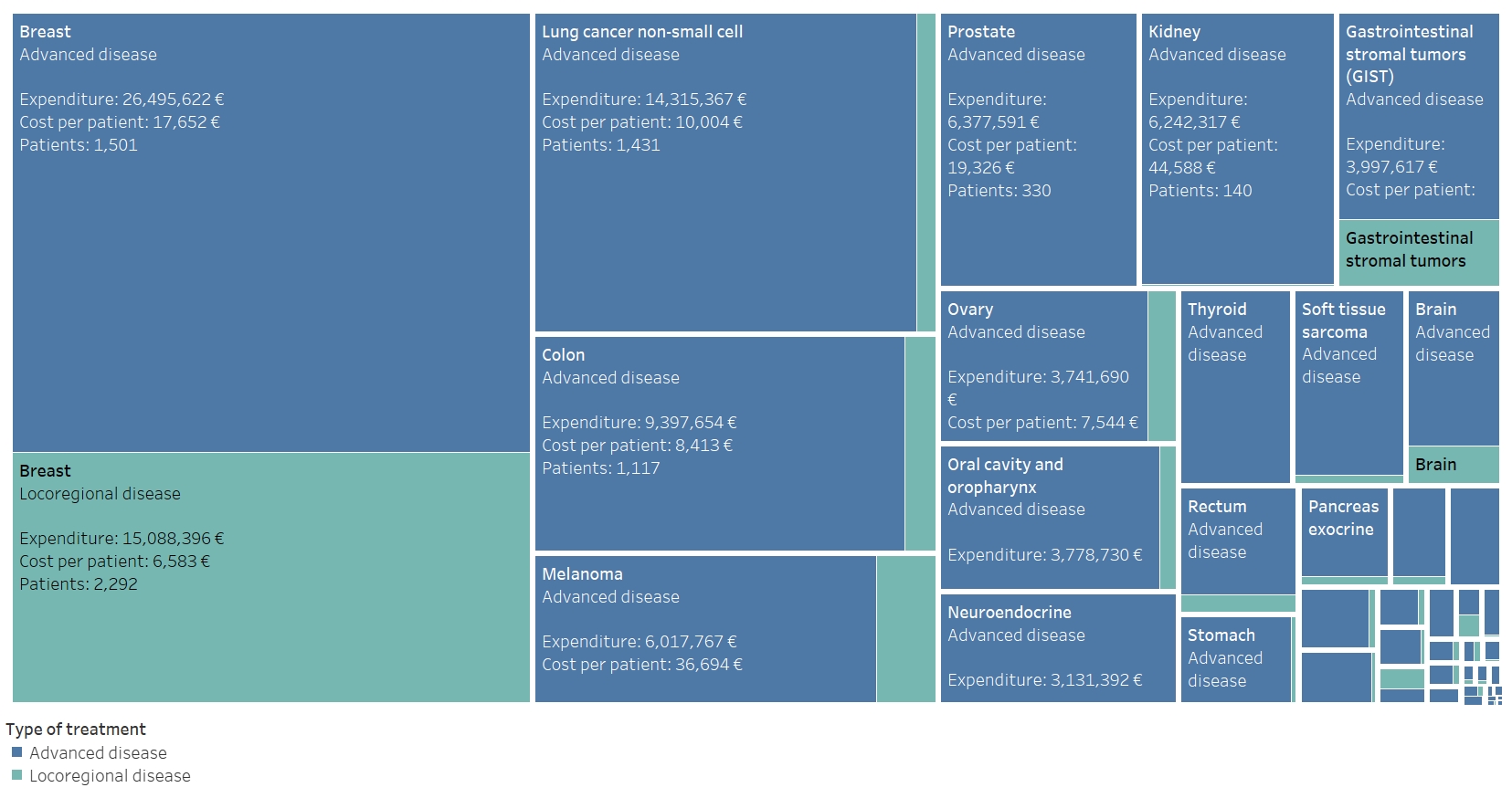

Supplement: Supplementary file 1 [file curroncol-30-00580-s001.zip › Supplementary Figure S2 revised.jpg]
